# Studying gender in the experiences of patients with heart failure: A scoping review of qualitative studies and methodological recommendations

**DOI:** 10.1177/17455057241305078

**Published:** 2025-01-30

**Authors:** Elias Thomas, Petra Verdonk, Jeanine Roeters-van Lennep, Hanneke Rhodius-Meester, Louis Handoko, Linda Schoonmade, Majon Muller, Maaike Muntinga

**Affiliations:** 1 Department of Internal Medicine – Geriatrics Section, Amsterdam UMC, Vrije Universiteit Amsterdam, Amsterdam, the Netherlands; 2 Department of Ethics Law and Humanities, Amsterdam UMC, Vrije Universiteit Amsterdam, Amsterdam, the Netherlands; 3 Amsterdam Public Health, Quality of Care, Amsterdam, the Netherlands; 4 Department of General Medicine, Erasmus Medical Center, Rotterdam, the Netherlands; 5 Alzheimer Center Amsterdam, Neurology, Vrije Universiteit Amsterdam, Amsterdam UMC location VUmc, Amsterdam, the Netherlands; 6 Department of Geriatric Medicine, The Memory Clinic, Oslo University Hospital, Oslo, Norway; 7 Department of Cardiology, Amsterdam UMC, Vrije Universiteit Amsterdam, Amsterdam, the Netherlands; 8 Medical Library, Vrije Universiteit Amsterdam, Amsterdam, the Netherlands; 9 Amsterdam Cardiovascular Science, Amsterdam, the Netherlands

**Keywords:** health equity, gender medicine, patient experience, heart failure, scoping review, intersectionality

## Abstract

**Background:**

Considering how gendered experiences play a role in the lives of patients with heart failure (HF) is critical in order to understand their experiences, optimise clinical care and reduce health inequalities.

**Objectives:**

The aim of our study was to review how gender is being studied in qualitative research in HF, specifically to (1) analyse how gender is conceptualised and applied in qualitative HF research; and (2) identify methodological opportunities to better understand the gendered experiences of patients with HF.

**Eligibility criteria:**

We conducted a systematic search of literature, including qualitive or mixed-methods articles focussing on patients’ perspectives in HF and using gender as a primary analytical factor, excluding articles published before 2000.

**Sources of evidence:**

Our search returned 3121 records, which were independently screened by two authors, resolving disagreements through a consensus procedure.

**Charting methods:**

Two reviewers extracted the characteristics of the included studies and methodological quality. We applied the *Integrating Sex and Gender Checklist* and gender theory as an analytical tool to synthesise results relating to the conceptualisation and application of gender in the included studies.

**Results:**

We included 11 qualitative articles that used interviews (*n* = 10) or focus groups (*n* = 1) to investigate the role of gender in experiences of patients with HF. None of the included studies defined their conceptual approach to gender, or used gender-related theoretical frameworks. This led to results and conclusions which were drawn along binary lines – representing gender as two separate, oppositional and mutually exclusive categories, and paying little attention to the dynamic, relational and context-dependent aspects of gender.

**Conclusions:**

Although researchers have investigated the role of gender in the experiences of patient with HF, methodological improvements are needed to prevent the current retelling of gender as a binary variable with two opposed and mutually exclusive categories. To better understand gendered experiences in HF, researchers need to avoid a reductionist and essentialist approach to gender. To this end, researchers should clearly state their conceptual approach to gender and analyse their findings using state-of-the-art gender theoretical frameworks and intersectional approaches. Ultimately, this will allow the development of tailored and effective clinical care.

## Introduction

Heart failure (HF) is a disease which places an enormous burden on patients worldwide and on the healthcare system.^1^ Increasingly, researchers have shown that sex-related variables (biological aspects of having a male or female body) and gender-related variables (societal norms, expectations and roles of women and men) play an important role in HF, including pathophysiology, diagnosis, allocation and efficacy of treatment, clinical outcome and daily living.^2,3^ This has led to the inclusion of sex and gender variables in HF research as good practice.^4–6^ Furthermore, research has shown the importance of considering gender and social context in the experiences of patients with chronic diseases, including HF.^7–10^ In this systematic scoping review, we critically investigate how gender is studied in qualitative HF research, and identify opportunities for methodological improvement.

Researchers have shown differences in terms of the clinical characteristics of HF between men and women. Men with chronic HF have a lower life expectancy compared to women, but women with HF generally have lower quality of life, more functional impairment, and a higher incidence of depressive symptoms than men.^11–15^ Despite increasing awareness of these differences, women are still under-represented in HF research, and efforts are being made to reduce the difference between inclusion rates for women and men in clinical trials.^16–18^ Historically, HF researchers have focused on the biological or clinical characteristics of women and men to explain differences in prognosis, disease impact and patient experiences, and some studies have shown that biological factors contribute to differences between men and women.^2,19^ This includes the influence of sex hormones, inflammation status, type of HF (e.g. women are more affected by HF with preserved ejection fraction than men) and the differential contribution of risk factors for HF.^19,20^ Furthermore, sex-specific aetiologies, such as the effects of pregnancy-related complications and premature menopause on development of HF, are increasingly being studied.^2,21^ These factors influence the clinical phenotypes of HF, as well as patient experiences and healthcare needs.

However, differences between men and women with HF are also affected by gender roles, gendered relations and institutional factors, including perceptions of health, presentation and responses to symptoms of illness, expectations about giving and receiving care and sexism in medicine.^10,19,22,23^ In current HF research, gender continues to be relatively neglected, and the terms *sex* and *gender* are used interchangeably, with a resulting lack of clarity about the relevance of sex versus gender.^2,10,24–26^ Especially in HF, where relations and social contexts shape patients’ perception of symptoms, disease burden, and access to (self-)care, a clear conceptualisation of gender is needed to reduce disparities in the domain of patient experience.^12,27^ In this systematic scoping review, we use gender theory to analyse how gender is considered in current qualitative HF research, and to identify methodological opportunities to improve insight in the everyday experiences of people living with HF. By assessing qualitative studies that investigate sex and gender in HF and highlight patient perspectives, we (1) examine critically how gender is conceptualised and applied in current qualitative HF research by using a gender theoretical framework; and (2) formulate methodological recommendations to analyse gender in qualitative research focusing on patients’ experiences in HF.

## Methods

We conducted a systematic scoping review to identify studies investigating gender and patients’ experiences in HF. Unlike systematic reviews, which aim to synthesise evidence for highly focused research questions, scoping reviews serve to assess the current body of evidence in the relevant scope of the question concerned and are particularly useful for identifying the type of evidence or approaches used in the current body of literature to approach research questions (*how* questions are answered).^28^ Our goal was not to perform a comprehensive literature overview of how exactly sex and gender impact the experiences of patients with HF, but rather to use current literature to explore how gender is conceptualised and applied when it is investigated in qualitative studies. Prior to conducting the study, we defined methodological strategies (such as inclusion and selection criteria and the analytical framework) in a study protocol; however, this protocol was not registered or published.

### Analytical framework: gender theory

We define ‘gender’ as the socially constructed roles, behaviours, expressions and identities of men, women and gender-diverse people.^19,29^ Gender includes economic relations, power relations, affective relations and symbolic relations that operate simultaneously at intrapersonal, interpersonal, institutional and society-wide levels.^30^ How gender is lived, understood and given meaning to is context-dependent: it might differ between sociocultural and personal contexts, and enactment of gender is negotiated by and among individuals, and in societies. It is therefore incorrect to reduce gender to binary and mutually exclusive categories (‘male–female differences’): it is a dynamic and relational sociocultural phenomenon and should be approached as such in clinical research. Furthermore, it is important to bear in mind that healthcare in itself is gendered: medical knowledge is considered ‘neutral’, while in fact it is largely based on the lives and bodies of White, cisgender and male individuals (‘referent man’), which has been the cause in institutionalised male bias.^31–33^ Furthermore, gender shapes health-seeking behaviour, the reporting of symptoms, access to care and the development of healthcare systems and policies.^34–38^ The WHO defines a healthcare system as ‘all organizations, people and actions whose primary intent is to promote, restore or maintain health’.^39^ However, gender even shapes what is perceived as health or healthy, and therefore is relevant when considering what constitutes good healthcare.^34,39^ Furthermore, gender is intertwined with other axes of social position, including race, class, sexuality and ability.^40^ In HF, for example, gendered and racialised experiences compound to create inequality within groups of women in terms of advanced therapy for HF, as well as experiences of care in private networks.^41,42^ Interplay between these structural inequalities might be understood through the concept of intersectionality, which takes the relationships and interplay between different social characteristics, including gender, race, class, age and sexuality, into account.^40,43–45^

### Positionality

An important element in critical and intersectional research methods is consideration of positionality of our research team because social locations and biases influence research questions and the interpretation of data.^46^ The authors are a group of individuals of different ages and genders (men, women and transgender individuals). As a team with diverse gender identities, we are more aware of unspoken and often unnoticed assumptions about gender. Specifically, being a transgender person (ET), and therefore operating outside of dominant gender paradigms brings an awareness which facilitates the identification of cis-normative assumptions in the studies in this review. Our team is relatively homogenous in terms of race and nationality: ET, PV, MMuller, HR, JRvL, LS and MMuntinga are White and Dutch. MLH’s ethnicity is Southeast Asian, born and raised in Western Europe. All of the authors mainly have experience with healthcare in a Western setting. Furthermore, all the authors received university education and work in an academic hospital. Our training as academic scholars may therefore have led to certain beliefs about what ‘good research’ is – such as using clear textual definitions for gender – which may not be shared by persons with HF who have other backgrounds shaping their knowledge. At the time of writing, ET is a medical doctor and PhD student, and MMuller and HR are medical specialists in geriatric medicine. MLH is a cardiologist specialised in HF, and JRvL is an internal medicine specialist with expertise in the role of sex in vascular disease. MMuntinga is a medical doctor and works as an assistant professor. PV is a psychologist and associate professor specialising in gender and diversity in health. Since most of the authors are medical doctors, PV’s views led to valuable insights from the perspective of a ‘medical outsider’, although this researcher has many years of experience working in an academic hospital as a researcher.

### Search strategy

This systematic scoping review followed the Preferred Reporting Items for Systematic reviews and Meta-Analyses extension for Scoping Reviews (PRISMA-ScR) Statement.^47,48^ Before performing and designing the search, key words were identified using knowledge of the research field as well as terms used in medical literature investigating either patients’ experiences in HF or the role of gender in clinical disease. After identifying relevant databases and consulting with an experienced librarian (LS), the primary author (ET) and LS collaborated to develop a comprehensive search of qualitative literature investigating gender in HF. This search was conducted in the bibliographic databases Medline (Ovid), Embase.com, Cinahl (EBSCOo), APA PsycINFO (EBSCO), the International Bibliography of Social Sciences (IBSS/ProQuest), Web of Science Core Collection and Scopus from inception to 15 March 2023. Search terms included both controlled terms and free-text terms, including ‘heart failure’ and ‘gender’ and ‘daily experiences’. In addition, we conducted a manual search and snowballing to identify additional relevant articles, and cross-referenced our findings with references from other articles investigating gender in HF. The full search strategies for all databases can be found in the Supplemental Information (Table S1).

### Selection process and extraction

We used the PRISMA-ScR checklist (Supplemental Table S3).^47^ Two reviewers (ET and MMuntinga) independently screened all potentially relevant titles and abstracts for eligibility using Rayyan.^49^ To ensure the topicality of our findings – given shifting societal norms and perceptions of gender – we excluded articles published before 2000. Gendered experiences inform the perception of illness and care,^27^ and focussing on patients’ perspectives in qualitative studies has been shown to improve the relevance and quality of clinical research.^50^ We therefore report on studies that explore patients’ narratives and perspectives regarding HF. We included articles that met the following additional criteria: (1) original peer-reviewed articles in English reporting on qualitative or mixed methods investigations; (2) using gender as a main analytical factor to explore the meaning and impact of HF experiences by reporting sex and/or gender differences or applying gender theory as a framework. No age limits or exclusion criteria in terms of clinical setting (e.g. primary healthcare, hospital care or community care) were applied. Articles were excluded if they (1) evaluated only a specific intervention such as heart transplants or telemonitoring methods and (2) were written from the perspective of healthcare professionals or caregivers. Furthermore, articles focusing solely on palliative care were excluded because we believe the subject of palliative care merits separate investigation. Disagreements in the screening of articles were resolved via consensus procedure between two authors and aided by other authors if needed. Data of the included articles were extracted by the authors (ET, MMuntinga) using *the Integrating Sex and Gender Checklist* developed by the Gender and Health Institute of the Canadian Institute of Health research: a checklist of items used to extract and synthesise data on sex and gender, and report outcomes.^51^ Furthermore, the quality of the included articles was assessed using the *JBI Critical Appraisal Checklist for critical and interpretative research.*^52^ We included articles regardless of the results of the quality appraisal, and we provide narrative results of the appraisal in the results section as well as the full appraisal using a Supplemental Table S2. After the first round of data extraction, in which we summarised study findings, we viewed the methodological approaches and interpretation of the results through a critical gender lens using the definition of gender given above in the introduction.^43,44^ We synthesised these results in a narrative fashion, identified methodological pitfalls related to gender through this critical gender lens and analysed them thematically; the decision about which themes to include in the results was reached through consensus.^53^

## Results

### Study characteristics and quality appraisal

**Figure 1.**
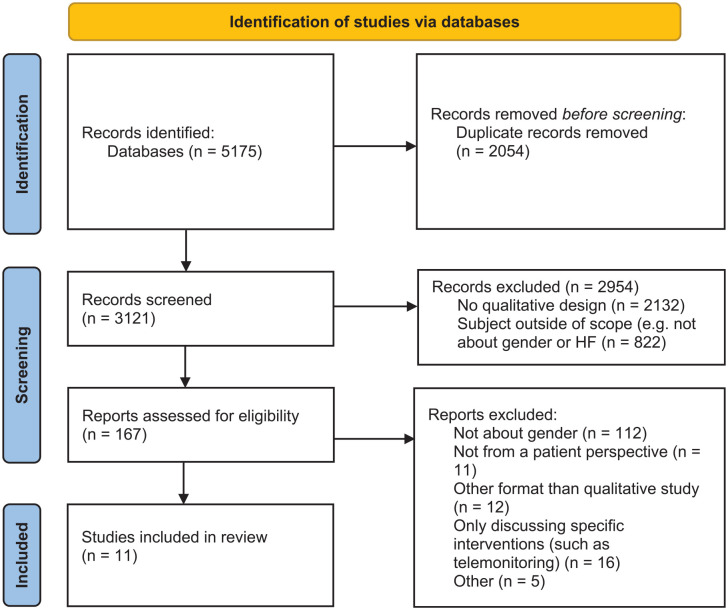
Flowchart of the search and selection procedure of studies.

Figure 1 shows the flowchart for the search and selection process.^54^ We identified a total of 11 studies that met our inclusion criteria (Table 1). These studies included interviews with a total of 172 patients from six different countries. Studies included participants aged 35–90 years old, and used in-depth,^55–57^ narrative^58,59^ or semi-structured^60–64^ interviews, semi-structured focus groups^65^ or mix-methods.^63^ Studies included only women (*n* = 5) or only men (*n* = 2), or both (*n* = 4). The main reasons given for studying sex and gender were to close the gender gap in the available data about HF and to improve women’s disadvantaged position in healthcare, reducing healthcare inequalities (Table 2). We appraised the quality of the included articles in terms of methodological rigour and found that, in almost all articles, the cultural and theoretical position of the researchers was not mentioned overlooking the ways in which social characteristics of the research team affect data gathering and data interpretation (Supplemental Table S2).^66,67^ The authors of only two studies reflected (briefly) on the influence of their own social position in relation to their research.^57,60^ Furthermore, there were incongruities in some studies between the research goal and the method used for data collection: the stated aim was to investigate differences between men and women with HF but the studies included homogenous samples of only women or only men.^56,58,63,65^

**Table 1.** Study characteristics of included articles.

S. No.	Author	Year	Country	Aim	Design and analytical approach	Study population
1	Evangelista et al.^55^	2001	US	To examine health perceptions of patients with HF and explore whether gender differences exist	Content analysis (not specified further) In-depth interviews	Thirty-two patients with a mean age of 52, with HF. The sample includes Whites (65%), African Americans (22%) and Asians (13%) *Patients with ‘major or acute psychological traumas’ were excluded (for example personal losses, substance abuse)*
2	Rhodes et al.^61^	2002	US	To examine and describe the experience of living with HF from the perspective of the women who live with HF.	Descriptive phenomenology (Husserl, 1950)**^68^** Semi-structured interviews (four interviews for each participant)	Four women, aged between 60 and 90 years, diagnosed with HF NYHA stage II. All Caucasian
3	Tyni-Lenné^62^	2004	Sweden	To gain insight from the patients’ perspective into how it is to live with moderate chronic HF	Qualitative inductive content analysis (Holsti, 1969)^69^ Semi-structured interviews	Twenty men, aged between 43 and 73 years, with HF NYHA II–III
4	Hägglund et al.^58^	2008	Sweden	To illuminate the lived experience of fatigue among elderly women with chronic heart failure	Qualitative content analysis (Graneheim and Lundman, 2004)^70^ Narrative interviews	Ten women, aged between 75 and 89 years, with HF NYHA III–IV
5	Allen et al.^63^	2009	US	To better understand the lived experience of women living with heart failure	Descriptive phenomenology (Husserl, 1950)^68^, Giorgi analytic method Semi-structured interviews	Four participants, aged between 49 and 64 years, with HF NYHA III
6	Sundin et al.^59^	2010	Sweden	Illuminate the meanings of support as it is experienced by elderly women with chronic HF	Phenomenological hermeneutic method of interpretation (Lindseth & Noorberg, 2004)^71^ Narrative interviews	Five women over 65 years old, with chronic HF NYHA III–IV
7	Riegel et al.^56^	2010	Australia	To describe HF self-care in men and women and to identify gender-specific barriers and facilitators to HF self-care	Analysed using cluster analysis In-depth interviews	Twenty-seven patients between 35–94 years old, with HF NYHA II–III
8	Burström et al.^65^	2012	Sweden	A deeper understanding of women’s experience of living with heart failure, focusing on security and insecurity	Qualitative content analysis (Graneheim & Lundman, 2004)^70^ Semi-structured focus group interviews	Eight women between 76 and 88 years old with HF NYHA II–III
9	Sano et al.^64^	2018	Japan	To identify self-management processes used by elderly male chronic HF patients	Constant comparative approach, with reference to the modified grounded theory approach by Kinoshia et al. (2007)^72^ Semi-structured interviews	Ten men between 70 and 85 years old, with HF NYHA II–III, who had not been hospitalised for the past 2 years
10	Surikova et al.^60^	2020	Canada	To gain a greater understanding of patient adjustment to CHF, to identify cultural and gender considerations that might present opportunities to improve selfcare adherence and quality of life.	Inductive qualitative approach with thematic content analysis Semi-structured interviews	Thirty patients between 18 and 75 years old wild HF NYHA I–III, identifying as Black, Chinese, South Asian, or Caucasian
11	Kamath et al.^57^	2021	India	To understand the principal factors affecting self-care among Indian chronic HF patients and propose that at the foundation are socially constructed processes rooted in cultural factors and realised in behavioural traits	Grounded theory analysis (Charmaz, 1990)^73^ In-depth interviews	Twenty-two patients (and caregivers) with a mean of 61 years old, with HF NYHA II–IV who also had a caregiver who could be interviewed. Purposive sampling was applied to recruit patients from different classes of socio-economic status, health literacy, medication adherence, and gender.

**Table 2.** Methodological considerations in relation to gender.

Author		Introduction		Methods and results				Discussion			
		Is gender defined by the authors, or are gender-related frameworks used?	What is the rationale behind the investigation of gender?	What is the social background of participants?	Are exclusion criteria used?	Is homogeneity within groups of men and women described?	Is the social background of the research team described?	Do the authors reflect on the social context to which their results are applicable?	Are the finding questioned from a gender perspective?	Are clinical recommendations given by the authors?	Are gaps in research identified?
1	Evangelista et al.^55^	No	Gender equity (women are underrepresented in clinical research, increasing number of women with HF)	- Men and women - Diverse ethnicity (Whites, African American, Asian) - Education (high school – college graduates) - Marital status (married vs not married) - Employment (employed vs retired)	Not-English-speaking participants Participants with “major physical pr psychological traumas in the previous 6 months”	No *Age is mentioned*	No	Yes *In terms of age*	No	Patient teaching can be tailored to address gender-specific concerns of men and women	Elucidate the relationship between meaning, spirituality, neuroticism, coping behaviours and health perceptions and psychosocial adjustment to HF
2	Rhodes et al.^61^	No	Gender equity (women are underrepresented in clinical research, but experience more disability)	- All women - All Caucasian - No other characteristics	Younger than 60, older than 90	No	No	No	No	Health personnel should encourage positive focus and empowerment in patients with HF to improve their physical and mental health	Investigate experience of patients with a recent HF diagnosis Longitudinal analysis
3	Europe and Tyni-Lenné^62^	No	Not specified	- All men - Employment (employed, retired because of age or illness)	No	Yes *Age is mentioned*	No	No	No	No	Impact of HF on ‘different type of patients’ Comparison of the experience of chronic HF between men and women
4	Hägglund et al.^58^	No	Male–female differences (to understand differences observed by scientific studies)	- All women - Living situation (alone vs together, type of home – all patients lived in an apartment)	Younger than 70, patients in nursing homes	Yes *Age is mentioned*	No	No	No	Nurses need to enable a dialogue concerning psychological and existential issues related to the experience of fatigue	Comparison with of illnesses in which patients experience fatigue should be made
5	Allen et al.^63^	No	Gender equality (women with heart failure report more disease burden than men, and are underrepresented in research)	- All women - Verbally articulate - Living situation (alone vs with others) - Education (high school and college graduates)	- Non-English speakers - Hearing impairment, cognitive impairment	Yes *the authors note that their sample is “relatively homogenous [. . .] and may not be transferable to [. . .] those who are older or have different ethnic or racial backgrounds*”	No	No	No	Women could be screened for depression.	Multi-site studies should be performed. Further exploration is needed to increase our knowledge of [*. . .*] the general population of women with HF.
6	Sundin et al.^59^	No	Male–female differences (in terms of roles, support, and life situation are present in literature)	All women	- Dementia, mental disability - Younger than 65	No	No	No	No	The authors emphasize the value of a confirming relationship during disease, but do not give concrete clinical advice	No
7	Riegel et al.^56^	No	Understanding male–female differences observed in scientific literature	- Men (*n* = 19) and women (*n* = 8) - Marital status - Living situation - Education (high school or university graduates) - Employment status (retired or not) - Financial status (‘have enough to make ends meet’)	- MMSE < 24 - Patients not fluent in English - Patients living in an extended care facility	No	No	No	No	Tailoring intervention approaches based on an assessment of attitudes and values [. . .] which may differ by gender	Interventions designed to build confidence and augment available support need to be tested. Family functioning should be measured. Research should be repeated in a larger sample with more variation in terms of marital status.
8	Burström et al.^65^	No	Understanding male–female differences observed in scientific literature	- All women - Age 76–88 years old - Living situation (alone versus together with spouse)	- Patients with ‘communication problems’	No	No	No	No	Understanding women’s outlook on the past, the present and the future to be able to enhance their feelings of security.	Women’s personal interpretations of living with HF focusing on security and insecurity
9	Sano et al.^64^	No	Not explicitely named	- All men - >65 years old - Living situation (alone or with family)	- Not able to communicatie in Japanese - Patients with cognitive impairment - Patients with terminal illnesses	No	No	No	No	Nurses should survey patients about their typical dietary and activity habits before hospitalisation [. . .] Advising patients how to modify and adapt self-care practices and activity.	Multisite studies and studies including women and younger patients with heart failure, should be performed.
10	Surikova et al.^60^	No	Health equity (for ethnic minorities as well as men and women)	- Men (20) and women (10) - Racial/ethnic background (Black, Chinese, South Asian, Caucasian) - Years of education	No	No	No *Although the authors do note that the team communicated in many languages, including English, Cantonese, Mandarin, Hindi and Punjabi*	Yes Authors note the heterosexual context of their findings	No	No	To develop and test culturally sensitive interventions that could facilitate self-care and improve quality of life among visible minorities living with HF. Sexual health in the context of quality of life for LGBTQI persons and women should be further explored
11	Kamath et al.^57^	No	Identifying gender-related beliefs and behaviours	- Men (13) and women (9) - Socioeconomic status (ranging from low to upper acc. to kuppuswamy’s scale) - Health literacy - Patients living in rural and urban areas	- Patients unable to provide coherent verbal information due to any medical reason - Patient without a principal caregiver	No	Yes ‘higher socioeconomic group and urban upbringing’	Yes	Yes Authors reflect on societal norms and expectations in relation to illness and ‘being sick’, as well as on gender roles in terms of responsibilities.	The authors of this paper developed a theory-based intervention package to improve HF self-care, which will be tested in a RCT. Assessing for and addressing sociocultural determinants of health during clinical interactions may help improve self-care, medication adherence and clinical outcomes	Future research should be carried out in the community (eg outside hospitals)

We identified two overlapping and interrelated themes: (1) defining and conceptualising gender; and (2) heterogeneity in gendered experiences. We elaborate on each theme below using examples from the results of the included studies.

#### Defining and conceptualising gender

We found that gender neither was defined or conceptualised in the included studies, nor it was explicitly stated whether researchers were investigating sex or gender, or both. None of the studies used gender-related theories or frameworks for the presentation of their aims or rationale, and none described how data relating to gender or sex were collected. Authors often used the phrase ‘male–female differences’ to refer to the influence of gender on patients’ experiences or healthcare needs. Furthermore, gender and sex were not explicitly distinguished in the aims, methods, results and discussions. For example, in a study examining fatigue in HF, no distinction was made between potential sex-based physiological differences leading to specific symptoms, or how aspects of identity and social context influence the burden and needs that arise for patients.^58^ One study group reflected on societal expectations relating to gender, explaining how societal views of women as family care-givers may lead to the relative neglect of their own health in HF.^57^ These authors also reflected on the conflation of sex-linked biology and gender in clinical practice, describing how cardiovascular risk behaviour in patients with HF – which is shaped by gendered roles and expectations (such as smoking or diet) – influences physiology.^57^

### Relational aspects of gender

The studies in our sample generally overlooked relational aspects of gender. Most of them described male–female differences in experiences of living with or coping with HF at the individual level. For example, many studies addressed differences between women and men in terms of disease self-management, and one study noted that men seemed to be more confident than women about adjusting their medication in line with HF symptoms.^56^ However, these studies did not theorise how these differences were produced by gendered factors at the institutional level, or how structural gender hierarchies played a role in individual experiences of being a man or a woman with HF. Furthermore, few studies investigated how gender was entangled with romantic relationships, family dynamics or other relationships of people with HF. Moreover, none of the studies discussed how gendered dynamics and differences might play a role in interactions with patients and their care providers (such as gender differences in the provision of information to patients, or trust in patient–physician relationships). The overlooking of gender as a relational phenomenon was further illustrated by the absence of descriptions of positionality statement of the researchers, as mentioned above.

### Normative assumptions about gender

In all studies, patients living with a spouse had – or were assumed to have – heterosexual relationships with a ‘traditional’ distribution of gender roles and responsibilities between these couples. When functional limitations due to HF were reported on, authors quoted men as finding physical decline hard to adapt to (‘*I’ve always been a big strong guy*’) or viewing paid employment as important (‘*work is really important to us men*’).^64^ In contrast, women were more often quoted as having difficulty performing household tasks.^55,56,58^ However, few studies reflected on how the emergence of the gendered distribution of roles and responsibilities, such as domestic tasks for women, is context-dependent, and may be different in individuals who do not comply with traditional living structures, such as non-heterosexual or non-cisgender individuals. Only one study noted the absence of non-heterosexual and non-cisgender patients as a limitation in the generalisability of their findings.^60^

#### Heterogeneity in gendered experiences

We also analysed the participants included by the authors, and how these voices were represented in the reporting of their findings. Social characteristics reported included living situation (described in five studies), education (*n* = 4), employment (*n* = 3), financial circumstances (*n* = 2), ethnicity (*n* = 3) and health literacy (*n* = 1). In general, the studies provided little description of the social background of their participants. Furthermore, where background was described, White, ‘highly-educated’, patients proved to be the majority. Of the three studies that described the ethnicity of their patients, two studies stated that they included patients of ethnic backgrounds other than White or Caucasian,^55,60^ and one study included Caucasian participants only.^61^ One study reflected on the absence of non-White participants in their study, stating that it was possible that their findings may not be applicable to other, non-White, populations.^63^ Purposeful sampling of patients was described by one study only, which explicitly stated that patients were recruited from backgrounds that were diverse in terms of health literacy, geography and socioeconomic status.^60^ Furthermore, most studies excluded non-English-speaking participants, patients with cognitive impairment, or patients with ‘communication problems’. One study also excluded patients with ‘major personal traumas’, including loss of a family member, or substance abuse. One study explicitly stated that they included patients who spoke different languages – using members of the research team as translators.^57^ No other studies referred to the use of interpreters/translators. Most researchers did not reflect on the relative homogeneity of participants. This was reflected in discussions of social isolation: several of the studies mentioned more social isolation among the interviewed women than among the men.^56,65^ However, the authors did not reflect on the social factors underlying social isolation such as geographical, cultural or socioeconomic differences. Furthermore, the relative homogeneity of the samples was often not taken into account when making clinical recommendations: only three studies stated that tailored, ‘gender-specific’ care was required.^55,63,65^ However, there was no discussion of how gender-specific care might be informed by care that takes other aspects of social identity – such as race or class – into account, nor did the authors reflect on the associated problems with the generalisability of their clinical recommendations.

## Critical gender analysis

We now turn to the analysis of the results of using theoretical gender and intersectionality frameworks and applying the analytical lens described in the methods section. We identify methodological shortcomings in current qualitative HF literature and discuss the implications of these shortcomings for research and clinical practice.

First of all, the studies we examined did not state whether sex, gender or both were being investigated. Conflating the concepts of sex and gender may result in essentialist narratives about the influence of gender on HF experiences.^74^ In HF, sex-related biological differences, as well as gender, affect illness and outcome. An a priori distinction between sex and gender, and a consideration of the ways the two concepts are interrelated, is imperative to understand the relevance and applicability of the two concepts for research and care.^19,26,29^ Furthermore, despite the good intentions evident in the included studies – understanding differences between men and women in HF and improving health equity – some studies investigated the role of gender by including women only. Gender was therefore assumed to be synonymous with women and women’s health, disregarding the impact of men’s and non-binary persons’ gendered experiences.^34,75^

Furthermore, women and men were often approached as separate and mutually exclusive categories, implying a simplistic and individualised view of gender and disregarding its contextual and contingent nature.^29,76^ The relational and dynamic aspects of gender – specifically the role of interactions with healthcare professionals and the healthcare system in general – received relatively little attention.^30^ By not reflecting on the relational aspect of gender, findings were presented as inherent (or *essentialist*^a^) characteristics of either men or women, disregarding gender relations at an interpersonal, societal and institutional level. This includes relationships with spouses and family members, with clinicians, and with the healthcare institution in a broader sense. In HF, there is ample evidence that these gendered relations, including social hierarchies and power dynamics in clinical care, are important in shaping patients’ experiences, treatment allocation and clinical outcome.^23,77–79^ Furthermore, concepts of masculinity and femininity in the context of health reflect intercultural differences, and the societal roles of, and expectations about, women and men change over time, and these dynamic aspects of gender were only briefly considered, if at all, in the studies we examined.^45^ This phenomenon has been extensively described in the context of health, and considering gender in this contextualised way could help researchers to deconstruct the tendency to perceive and present differences between women and men as inherent, constant and unchangeable.^30,80,81^

The assumption of fixed ‘gender-related traits’, in which a feminine truth is opposed to a masculine truth, was further reinforced in the recommendations: some authors called for the tailoring of care to the specific needs of women and men.^56^ a portrayal of male and female realities as inherently different and mutually exclusive might reproduce stereotypical notions of masculine and feminine social roles, which does not do justice to the complexity of living with heart failure as a man or a women and could perpetuate biased assumptions about people’s experiences. Such biases might trickle down into clinical care, where they might manifest as care inequities.^35,82^ For example, some authors recommended ‘gender-specific’ approaches, recommending more focus on emotional support for women with HF. Although it may be true that the included studies found more examples of women discussing their emotional world than men, none of the authors questioned how these differences in narratives arose. Individual, interpersonal and societal expectations relating to masculinity make it more challenging for many men to discuss problems relating to mental health, even though depression and anxiety are also present in men with HF and lead to adverse outcomes.^34,83^ Furthermore, social hierarchies and power dynamics between researchers, physicians and patients have an effect on data gathering and data interpretation – involving gender as well as race and class.^23,77^ However, few research teams considered their own social background. This reflects an assumption of researchers – and physicians – that they are gender-neutral, ascribing findings solely to the characteristics of participants and disregarding their own role in the data collection and interpretation process.^36,38^ There is convincing evidence that scientists and science are not neutral: gender bias in research, as well as other forms of social bias, determine which research questions are asked, how they are asked and answered and how results are interpreted and disseminated, reproducing and entrenching health inequalities.^37,45^ Furthermore, these social biases are also present and highly relevant in clinical practice, creating and perpetuating health inequalities.^35,84,85^ For example, women’s cardiovascular risk is still often underestimated in clinical practice, leading to inadequate cardiovascular preventive care for women as a group.^35,86^ Reflecting on these biases is therefore urgently needed to promote social equality in HF.

Furthermore, we found little reflection in the included studies on the variability *within* groups of men or women. Because the majority of patients in the studies in our review were White, heterosexual and relatively highly educated, there was an imbalance in the included narratives in which women or men who do not fall into these categories are under-represented. This includes groups who are most susceptible to the development of HF and to adverse outcomes, such as African–American women or women classified as having ‘low socioeconomic status’.^87,88^ Especially in HF, where home-based self-care is a cornerstone of disease management, considering the different aspects of patients’ social context is crucial to understanding experiences in order to tailor clinical care accordingly.^89^ In the example of the differences in social isolation between men and women which were found in the included studies, more nuanced conclusions could be possible if social factors other than gender are considered. Studies in HF have shown that other social variables such as geographical (living in a rural or an urban environment) or cultural differences (e.g. living in one home with children or other family, as opposed to living alone), as well as racist and classist experiences, are also related to social isolation.^90,91^ A lack of intersectional representation in the included studies as well as the binary conceptualisation of gender observed in this review may have led to conclusions which overlook the intricate ways in which gender is linked with other social and cultural variables.^92,93^ This limited the generalisability of findings – in clinical practice these social variables never exist in isolation but instead are intertwined with other social positions such as race and class – and serves to maintain healthcare inequalities for minorities.^40,45,93^ Qualitative research involving patients with these intersecting identities, and researchers reflecting on these intersections, is needed to enable us to better understand the variety of lived realities of patients with HF.

## Discussion

In this systematic scoping review, we critically examined how gender is conceptualised and applied in qualitative studies into the experiences of people with HF, with the aim of identifying methodological opportunities to improve the understanding of patients gendered experiences through qualitative research. We found that, although there were several studies investigating the role of gender in HF, an improvement in methodological quality is needed to better understand the relationship between gender and the experiences of patients with HF. Using a critical gender lens, we have shown that studies did not take the analysis of gender further than a binary conceptualisation, presenting gender as an inherent or fixed trait instead of a sociocultural phenomenon which is dynamic and relational in nature.^30,83^ Women and men were presented as homogenous groups, overlooking intersectional aspects of gendered experiences.^45^ Furthermore, research and medicine were approached as gender-neutral, and there was a paucity of reflection on gender-related biases and assumptions amongst researchers, physicians, and the medical system in general. Although we did not perform a comprehensive overview of all literature in HF and therefore may have overlooked studies that did address gender in heart failure from a more complex perspective, we conclude based on our findings that the majority of current studies overlook the importance of relational, dynamic and intersectional dimensions of gender. An oversimplified conceptualisation of gender might inform the reproduction of normative and essentialist assumptions about men and women, inadequately portray gendered lives of patients with HF as uncomplicated, and perpetuate healthcare inequalities through socio-clinical bias. We discuss the limitations of this study below and make several recommendations to improve the methodological quality of studies investigating gender in HF experiences.

### Limitations

This study is a scoping review; therefore, it may not have included all available studies investigating gender in HF. Furthermore, the number of studies in this review are relatively small, and limited to the context of chronic HF. Future studies should focus on gathering more data – both in – and outside the context of HF – to assess the robustness of our findings. Furthermore, our study focussed on patient narratives, not assessing the perspectives of patients’ loved ones, informal care givers and clinical researchers. Since gender is shaped and performed in relation with others, including the perspectives of these others might further our understanding of how conceptualisations and preconceptions of gender might shape patients’ lives and clinical outcomes. Furthermore, the majority of the included studies were set in Western or European countries, limiting the validity of our findings to other setting. Future research should focus on non-Western countries to assess differences in conceptualisation and application of genders in these settings, and reflect on lessons to be learnt from the differences and similarities in how gender is researched in different cultural settings.

### Recommendations for research

Firstly, in order for clinical research to lead to a better understanding of gender and health, and reduce healthcare inequalities, state-of-the-art theoretical frameworks should be used to define gender, and to theorise its relationship with health on a personal, interpersonal, institutional and societal level.^30,34,94^ These theoretical frameworks should include critical and intersectional approaches to gender.^40,43,81^ This is true for quantitative as well as qualitative studies, and although we stress the importance of applying of gender theory and intersectionality in quantitative studies, this is outside the scope of this study.^45^ Resources to improve the conceptualisation, application and critical reflection of gender in qualitative healthcare research include theoretical discussions, as well as practical articles and toolkits providing guidance in designing, executing and interpreting qualitative research.^40,43–45,51,95–97^ Studies in public health have already shown how the understanding and application of this knowledge might provide important opportunities to improve not only research but also health and clinical outcome for patients with HF.^98,99^ In addition, researchers and clinicians should be aware of their own gender-based biases and assumptions, and how they influence patient experiences, healthcare research and clinical care.^100–102^ Theoretical and practical guidance on the implications of social dynamics and power relations between participants, researchers and clinicians might be aided by applying the concept of *reflexivity* on the basis of the work of scholars theorising that concept in the context of health and healthcare research, and by performing multidisciplinary research in collaboration with social scientists.^46,103,104^

## Conclusion

In conclusion, we have identified potential methodological improvements in current studies investigating patients’ experience in HF by theorising gender as a relational and dynamic social phenomenon, considering intersectionality and reflecting on personal and institutional gender bias in research and medicine. These methodological improvements are crucial to prevent the reproducing of normative assumptions surrounding gender, and for qualitative research to achieve its aim of better understanding patients’ gendered experiences and ultimately reducing gender-based inequalities in research and clinical care.

## Supplemental Material

sj-docx-1-whe-10.1177_17455057241305078 – Supplemental material for Studying gender in the experiences of patients with heart failure: A scoping review of qualitative studies and methodological recommendations Supplemental material, sj-docx-1-whe-10.1177_17455057241305078 for Studying gender in the experiences of patients with heart failure: A scoping review of qualitative studies and methodological recommendations by Elias Thomas, Petra Verdonk, Jeanine Roeters-van Lennep, Hanneke Rhodius-Meester, Louis Handoko, Linda Schoonmade, Majon Muller and Maaike Muntinga in Women's Health

## Declarations

**Ethics approval and consent to participate:** Not applicable.

**Consent for publication:** Not applicable.

**Availability of data and materials:** The data of this study are available upon request from the corresponding author (ET).

## Notes

a. (Gender) Essentialism: The belief that women and men are fundamentally different in their capacities, interests and skills. In this view, biological sex is considered to be the primary factor determining gender.
