# Supplementary material for: Studying gender in the experiences of patients with heart failure: A scoping review of qualitative studies and methodological recommendations
Source: Womens Health (Lond). 2025 Jan 30;21:17455057241305078. doi: 10.1177/17455057241305078 (PMC11783506; doi:10.1177/17455057241305078)
Supplement: sj-docx-1-whe-10.1177_17455057241305078 – Supplemental material for Studying gender in the experiences of patients with heart failure: A scoping review of qualitative studies and methodological recommendations [file sj-docx-1-whe-10.1177_17455057241305078.docx]

**Supplementary information S2**

## Medline (Ovid) History and Search Details March 15, 2023

| **Search** | **Query** | **Results** |
| --- | --- | --- |
| #1 | Exp Heart Failure/ OR ("heart failure*" OR "cardiac failure*" OR "heart decompensation*" OR "myocardial failure*" OR "heart insuffic*" OR "myocardial insuffic*" OR "decompensatio cordis" OR HfpEF OR HfrEF OR "paroxysmal dyspnea" OR "cardiac asthma*" OR "cardiac edema*" OR "cardiac oedema*" OR "cardiac decompensation" OR cadiomyopath* OR "diastolic dysfunct*" OR "systolic dysfunct*").ti,ab,kf | 270,418 |
| #2 | Exp Intersectional Framework/ OR exp Sex Factors/ OR exp Gender Identity/ OR exp Sexual and Gender Minorities/ OR exp Sex Characteristics/ OR (intersectional* OR gender OR "sex diff*" OR "sex inequalit*" OR "sex bias" OR "sex-specific" OR "sex factor*" OR "sex based" OR "sex disparit*" OR "sex factor*" OR "sex characteristic*" OR "sex related" OR sexe* OR "male versus female" OR "men versus women" OR "men and women" OR "women and men" OR "male and female" OR "female and male" OR "female identit*" OR "male identit*").ti,ab,kf OR (women OR woman OR female OR man OR men OR male).ti | 1,299,690 |
| #3 | Attitude/ OR (satisf* OR perspective* OR perception* OR experienc* OR narrative* OR wish* OR desire* OR need* OR attitude* OR stories OR story OR account* OR challeng* OR problem* OR barrier* OR obstacle* OR concern* OR belief* OR believ* OR consideration* OR considering OR well-being OR meaning OR "care goal*" OR (patient* ADJ2 goal*) OR dignity OR "meaning-making*").ti,ab,kf | 7,842,926 |
| #4 | Exp Qualitative Research/ OR exp Focus Groups/ OR exp Interview/ OR exp Interviews as Topic/ OR exp Narration/ OR exp Personal Narratives as Topic/ OR exp Grounded Theory/ OR exp Observational Studies as Topic/ OR exp Observational Study/ OR exp Tape Recording/ OR ("thematic analys*" OR "content analys*" OR "focus group*" OR ethnograph* OR ethnograf* OR etnograf* OR photovoic* OR "field stud*" OR fieldwork* OR "field work*" OR phenomenolog* OR narration* OR narrative OR qualitative OR multimethodolog* OR "mixed method*" OR observation* OR "grounded theory" OR "audio recording*" OR "tape recording*" OR audiotape* OR ((semi-structured OR semistructured OR unstructured OR informal OR in-depth OR indepth OR face-to-face OR structured OR guide*) AND (interview* OR discussion* OR questionnaire*))).ti,ab,kf | 1,779,921 |
| #4 | #1 AND #2 AND #3 AND #4 | 443 |

## Embase.com History and Search Details March 15, 2023

| **Search** | **Query** | **Results** |
| --- | --- | --- |
| #1 | 'heart failure'/de OR 'forward heart failure'/de OR 'congestive heart failure'/exp OR 'diastolic dysfunction'/exp OR 'systolic dysfunction'/exp OR ('heart failure*' OR 'cardiac failure*' OR 'heart decompensation*' OR 'myocardial failure*' OR 'heart insuffic*' OR 'myocardial insuffic*' OR 'decompensatio cordis' OR HfpEF OR HfrEF OR 'paroxysmal dyspnea' OR 'cardiac asthma*' OR 'cardiac edema*' OR 'cardiac oedema*' OR 'cardiac decompensation' OR cadiomyopath*):ti,ab,kw | 530,803 |
| #2 | 'intersectionality'/exp OR 'sex difference'/exp OR 'sexual and gender minority'/exp OR 'gender identity'/exp OR (intersectional* OR gender OR 'sex diff*' OR 'sex inequalit*' OR 'sex bias' OR 'sex-specific' OR 'sex factor*' OR 'sex based' OR 'sex disparit*' OR 'sex factor*' OR 'sex characteristic*' OR 'sex related' OR sexe* OR 'male versus female' OR 'men versus women' OR 'men and women' OR 'women and men' OR 'male and female' OR 'female and male' OR 'female identit*' OR 'male identit*'):ti,ab,kw OR (women OR woman OR female OR man OR men OR male):ti | 2,032,047 |
| #3 | 'attitude'/de OR 'daily life activity'/exp OR 'quality of life'/de OR (satisf* OR perspective* OR perception* OR experienc* OR narrative* OR wish* OR desire* OR need* OR attitude* OR stories OR story OR account* OR challeng* OR problem* OR barrier* OR obstacle* OR concern* OR belief* OR believ* OR consideration* OR considering OR well-being OR meaning OR 'care goal*' OR (patient* NEAR/2 goal*) OR dignity OR 'meaning-making*'):ti,ab,kw | 10,531,831 |
| #4 | #1 AND #2 AND #3 | 12,116 |
| #5 | 'qualitative research'/exp OR 'interview'/exp OR 'narrative'/exp OR 'storytelling'/exp OR 'grounded theory'/exp OR 'observational study'/exp OR 'recording'/exp OR 'thematic analysis'/exp OR 'content analysis'/exp OR 'ethnographic research'/exp OR 'field study'/exp OR 'participant observation'/exp OR 'phenomenology'/exp OR 'qualitative analysis'/exp OR 'qualitative methods'/exp OR 'in depth interview'/exp OR 'face to face interview'/exp OR ('thematic analys*' OR 'content analys*' OR 'focus group*' OR ethnograph* OR ethnograf* OR etnograf* OR photovoic* OR 'field stud*' OR fieldwork* OR 'field work*' OR phenomenolog* OR narration* OR narrative OR qualitative OR multimethodolog* OR 'mixed method*' OR observation* OR 'grounded theory' OR 'audio recording*' OR 'tape recording*' OR audiotape* OR (('semi-structured' OR semistructured OR unstructured OR informal OR 'in-depth' OR indepth OR 'face-to-face' OR structured OR guide*) AND (interview* OR discussion* OR questionnaire*))):ti,ab,kw | 2,564,462 |
| #5 | #1 AND #2 AND #3 AND #4 | 1,600 |
| #6 | #5 NOT ('conference abstract'/it OR 'conference review'/it) | 930 |

## PsycInfo (Ebsco) History and Search Details March 15, 2023

| **Search** | **Query** | **Results** |
| --- | --- | --- |
| S1 | TI ("heart failure*" OR "cardiac failure*" OR "heart decompensation*" OR "myocardial failure*" OR "heart insuffic*" OR "myocardial insuffic*" OR "decompensatio cordis" OR HfpEF OR HfrEF OR "paroxysmal dyspnea" OR "cardiac asthma*" OR "cardiac edema*" OR "cardiac oedema*" OR "cardiac decompensation" OR cadiomyopath* OR "diastolic dysfunct*" OR "systolic dysfunct*") OR AB ("heart failure*" OR "cardiac failure*" OR "heart decompensation*" OR "myocardial failure*" OR "heart insuffic*" OR "myocardial insuffic*" OR "decompensatio cordis" OR HfpEF OR HfrEF OR "paroxysmal dyspnea" OR "cardiac asthma*" OR "cardiac edema*" OR "cardiac oedema*" OR "cardiac decompensation" OR cadiomyopath* OR "diastolic dysfunct*" OR "systolic dysfunct*") OR KW ("heart failure*" OR "cardiac failure*" OR "heart decompensation*" OR "myocardial failure*" OR "heart insuffic*" OR "myocardial insuffic*" OR "decompensatio cordis" OR HfpEF OR HfrEF OR "paroxysmal dyspnea" OR "cardiac asthma*" OR "cardiac edema*" OR "cardiac oedema*" OR "cardiac decompensation" OR cadiomyopath* OR "diastolic dysfunct*" OR "systolic dysfunct*") | 4,708 |
| S2 | DE ("Human Sex Differences" OR "Intersectionality" OR "Gender Identity" OR "Sexual Minority Groups") OR TI (intersectional* OR gender OR "sex diff*" OR "sex inequalit*" OR "sex bias" OR "sex-specific" OR "sex factor*" OR "sex based" OR "sex disparit*" OR "sex factor*" OR "sex characteristic*" OR "sex related" OR sexe* OR "male versus female" OR "men versus women" OR "men and women" OR "women and men" OR "male and female" OR "female and male" OR "female identit*" OR "male identit*" OR (women OR woman OR female OR man OR men OR male) OR AB (intersectional* OR gender OR "sex diff*" OR "sex inequalit*" OR "sex bias" OR "sex-specific" OR "sex factor*" OR "sex based" OR "sex disparit*" OR "sex factor*" OR "sex characteristic*" OR "sex related" OR sexe* OR "male versus female" OR "men versus women" OR "men and women" OR "women and men" OR "male and female" OR "female and male" OR "female identit*" OR "male identit*") OR KW (intersectional* OR gender OR "sex diff*" OR "sex inequalit*" OR "sex bias" OR "sex-specific" OR "sex factor*" OR "sex based" OR "sex disparit*" OR "sex factor*" OR "sex characteristic*" OR "sex related" OR sexe* OR "male versus female" OR "men versus women" OR "men and women" OR "women and men" OR "male and female" OR "female and male" OR "female identit*" OR "male identit*") | 1,805,051 |
| S3 | DE ("Attitudes" OR "Female Attitudes" OR "Male Attitudes" OR "Physical Illness (Attitudes Toward)") OR "Activities of Daily Living" OR "Quality of Life" OR "Health Related Quality of Life") OR TI (satisf* OR perspective* OR perception* OR experienc* OR narrative* OR wish* OR desire* OR need* OR attitude* OR stories OR story OR account* OR challeng* OR problem* OR barrier* OR obstacle* OR concern* OR belief* OR believ* OR consideration* OR considering OR well-being OR meaning OR "care goal*" OR (patient* N2 goal*) OR dignity OR "meaning-making*") OR AB (satisf* OR perspective* OR perception* OR experienc* OR narrative* OR wish* OR desire* OR need* OR attitude* OR stories OR story OR account* OR challeng* OR problem* OR barrier* OR obstacle* OR concern* OR belief* OR believ* OR consideration* OR considering OR well-being OR meaning OR "care goal*" OR (patient* N2 goal*) OR dignity OR "meaning-making*") OR KW (satisf* OR perspective* OR perception* OR experienc* OR narrative* OR wish* OR desire* OR need* OR attitude* OR stories OR story OR account* OR challeng* OR problem* OR barrier* OR obstacle* OR concern* OR belief* OR believ* OR consideration* OR considering OR well-being OR meaning OR "care goal*" OR (patient* N2 goal*) OR dignity OR "meaning-making*") | 3,017,530 |
| S4 | DE ("Qualitative Methods" OR "Qualitative Measures" OR "Grounded Theory" OR "Focus Group" OR "Focus Group Interview" OR "Phenomenology" OR "Interpretative Phenomenological Analysis" OR "Narrative Analysis" OR "Semi-Structured Interview" OR "Thematic Analysis" OR "Mixed Methods Research" OR "Observation Methods" OR "Direct Observation" OR "Participant Observation" OR "Interviews" OR "Semi-Structured Interview" OR "Phenomenology" OR "Interpretative Phenomenological Analysis" OR "Ethnography" OR "Audiotapes" OR "Videotapes" OR "Mixed Methods Research") OR TI (“thematic analys*” OR “content analys*” OR “focus group*” OR ethnograph* OR ethnograf* OR etnograf* OR photovoic* OR “field stud*” OR fieldwork* OR "field work*" OR phenomenolog* OR narration* OR narrative OR qualitative OR multimethodolog* OR “mixed method*” OR observation* OR “grounded theory” OR “audio recording*” OR “tape recording*” OR audiotape* OR ((“semi-structured” OR semistructured OR unstructured OR informal OR “in-depth” OR indepth OR “face-to-face” OR structured OR guide*) AND (interview* OR discussion* OR questionnaire*))) OR AB (“thematic analys*” OR “content analys*” OR “focus group*” OR ethnograph* OR ethnograf* OR etnograf* OR photovoic* OR “field stud*” OR fieldwork* OR "field work*" OR phenomenolog* OR narration* OR narrative OR qualitative OR multimethodolog* OR “mixed method*” OR observation* OR “grounded theory” OR “audio recording*” OR “tape recording*” OR audiotape* OR ((“semi-structured” OR semistructured OR unstructured OR informal OR “in-depth” OR indepth OR “face-to-face” OR structured OR guide*) AND (interview* OR discussion* OR questionnaire*))) OR KW (“thematic analys*” OR “content analys*” OR “focus group*” OR ethnograph* OR ethnograf* OR etnograf* OR photovoic* OR “field stud*” OR fieldwork* OR "field work*" OR phenomenolog* OR narration* OR narrative OR qualitative OR multimethodolog* OR “mixed method*” OR observation* OR “grounded theory” OR “audio recording*” OR “tape recording*” OR audiotape* OR ((“semi-structured” OR semistructured OR unstructured OR informal OR “in-depth” OR indepth OR “face-to-face” OR structured OR guide*) AND (interview* OR discussion* OR questionnaire*))) | 675,258 |
| S5 | S1 AND S2 AND S3 AND S4 | 364 |

## Cinahl (Ebsco) History and Search Details March 15, 2023

| **Search** | **Query** | **Results** |
| --- | --- | --- |
| S1 | MH "Heart Failure+" OR TI ("heart failure*" OR "cardiac failure*" OR "heart decompensation*" OR "myocardial failure*" OR "heart insuffic*" OR "myocardial insuffic*" OR "decompensatio cordis" OR HfpEF OR HfrEF OR "paroxysmal dyspnea" OR "cardiac asthma*" OR "cardiac edema*" OR "cardiac oedema*" OR "cardiac decompensation" OR cadiomyopath* OR "diastolic dysfunct*" OR "systolic dysfunct*") OR AB ("heart failure*" OR "cardiac failure*" OR "heart decompensation*" OR "myocardial failure*" OR "heart insuffic*" OR "myocardial insuffic*" OR "decompensatio cordis" OR HfpEF OR HfrEF OR "paroxysmal dyspnea" OR "cardiac asthma*" OR "cardiac edema*" OR "cardiac oedema*" OR "cardiac decompensation" OR cadiomyopath* OR "diastolic dysfunct*" OR "systolic dysfunct*") | 81,970 |
| S2 | MH ("Intersectionality" OR "Sex Factors" OR "Gender Role+" OR "Gender Bias" OR "Gender Identity+" OR "Sexual and Gender Minorities+" OR "Gender Specific Care") OR TI (intersectional* OR gender OR "sex diff*" OR "sex inequalit*" OR "sex bias" OR "sex-specific" OR "sex factor*" OR "sex based" OR "sex disparit*" OR "sex factor*" OR "sex characteristic*" OR "sex related" OR sexe* OR "male versus female" OR "men versus women" OR "men and women" OR "women and men" OR "male and female" OR "female and male" OR "female identit*" OR "male identit*" OR (women OR woman OR female OR man OR men OR male) OR AB (intersectional* OR gender OR "sex diff*" OR "sex inequalit*" OR "sex bias" OR "sex-specific" OR "sex factor*" OR "sex based" OR "sex disparit*" OR "sex factor*" OR "sex characteristic*" OR "sex related" OR sexe* OR "male versus female" OR "men versus women" OR "men and women" OR "women and men" OR "male and female" OR "female and male" OR "female identit*" OR "male identit*") | 2,876,138 |
| S3 | MH ("Attitude" OR "Attitude to Illness+" OR "Activities of Daily Living+" OR "Quality of Life") OR TI (satisf* OR perspective* OR perception* OR experienc* OR narrative* OR wish* OR desire* OR need* OR attitude* OR stories OR story OR account* OR challeng* OR problem* OR barrier* OR obstacle* OR concern* OR belief* OR believ* OR consideration* OR considering OR well-being OR meaning OR "care goal*" OR (patient* N2 goal*) OR dignity OR "meaning-making*") OR AB (satisf* OR perspective* OR perception* OR experienc* OR narrative* OR wish* OR desire* OR need* OR attitude* OR stories OR story OR account* OR challeng* OR problem* OR barrier* OR obstacle* OR concern* OR belief* OR believ* OR consideration* OR considering OR well-being OR meaning OR "care goal*" OR (patient* N2 goal*) OR dignity OR "meaning-making*") | 2,371,820 |
| S4 | MH ("Qualitative Studies+" OR "Thematic Analysis" OR "Content Analysis" OR "Focus Groups" OR "Ethnographic Research" OR "Field Studies" OR "Narratives+" OR "Multimethod Studies" OR "Observational Methods+" OR "Audiorecording" OR "Semi-Structured Interview" OR "Unstructured Interview") OR TI (“thematic analys*” OR “content analys*” OR “focus group*” OR ethnograph* OR ethnograf* OR etnograf* OR photovoic* OR “field stud*” OR fieldwork* OR "field work*" OR phenomenolog* OR narration* OR narrative OR qualitative OR multimethodolog* OR “mixed method*” OR observation* OR “grounded theory” OR “audio recording*” OR “tape recording*” OR audiotape* OR ((“semi-structured” OR semistructured OR unstructured OR informal OR “in-depth” OR indepth OR “face-to-face” OR structured OR guide*) AND (interview* OR discussion* OR questionnaire*))) OR AB (“thematic analys*” OR “content analys*” OR “focus group*” OR ethnograph* OR ethnograf* OR etnograf* OR photovoic* OR “field stud*” OR fieldwork* OR "field work*" OR phenomenolog* OR narration* OR narrative OR qualitative OR multimethodolog* OR “mixed method*” OR observation* OR “grounded theory” OR “audio recording*” OR “tape recording*” OR audiotape* OR ((“semi-structured” OR semistructured OR unstructured OR informal OR “in-depth” OR indepth OR “face-to-face” OR structured OR guide*) AND (interview* OR discussion* OR questionnaire*))) | 626,385 |
| S5 | S1 AND S2 AND S3 AND S4 | 1,647 |

## Web of Science Core Collection History and Search Details March 15, 2023

| **Search** | **Query** | **Results** |
| --- | --- | --- |
| #1 | TS = ("heart failure*" OR "cardiac failure*" OR "heart decompensation*" OR "myocardial failure*" OR "heart insuffic*" OR "myocardial insuffic*" OR "decompensatio cordis" OR "HfpEF" OR "HfrEF" OR "paroxysmal dyspnea" OR "cardiac asthma*" OR "cardiac edema*" OR "cardiac oedema*" OR "cardiac decompensation" OR cadiomyopath* OR "diastolic dysfunct*" OR "systolic dysfunct*") | 323,697 |
| #2 | TS = (intersectional* OR "gender" OR "sex diff*" OR "sex inequalit*" OR "sex bias" OR "sex-specific" OR "sex factor*" OR "sex based" OR "sex disparit*" OR "sex factor*" OR "sex characteristic*" OR "sex related" OR sexe* OR "male versus female" OR "men versus women" OR "men and women" OR "women and men" OR "male and female" OR "female and male" OR "female identit*" OR "male identit*") OR TI=("women" OR "woman" OR "female" OR "man" OR "men" OR "male") | 2,057,388 |
| #3 | TS= (satisf* OR perspective* OR perception* OR experienc* OR narrative* OR wish* OR desire* OR need* OR attitude* OR stories OR story OR account* OR challeng* OR problem* OR barrier* OR obstacle* OR concern* OR belief* OR believ* OR consideration* OR considering OR well-being OR meaning OR "care goal*" OR (patient* NEAR/2 goal*) OR dignity OR "meaning-making*") | 19,323,106 |
| #4 | TS = ("thematic analys*" OR "content analys*" OR "focus group*" OR ethnograph* OR ethnograf* OR etnograf* OR photovoic* OR "field stud*" OR fieldwork* OR "field work*" OR phenomenolog* OR narration* OR narrative OR qualitative OR multimethodolog* OR "mixed method*" OR observation* OR "grounded theory" OR "audio recording*" OR "tape recording*" OR audiotape* OR ((semi-structured OR semistructured OR unstructured OR informal OR in-depth OR indepth OR face-to-face OR structured OR guide*) AND (interview* OR discussion* OR questionnaire*))) | 3,372,115 |
| #5 | #1 AND #2 AND #3 AND #4 | 751 |

## Scopus History and Search Details March 15, 2023

| **Search** | **Query** | **Results** |
| --- | --- | --- |
| #1 | TITLE-ABS-KEY ("heart failure*" OR "cardiac failure*" OR "heart decompensation*" OR "myocardial failure*" OR "heart insuffic*" OR "myocardial insuffic*" OR {decompensatio cordis} OR {HfpEF} OR {HfrEF} OR {paroxysmal dyspnea} OR "cardiac asthma*" OR "cardiac edema*" OR "cardiac oedema*" OR {cardiac decompensation} OR cadiomyopath* OR "diastolic dysfunct*" OR "systolic dysfunct*") | 409,765 |
| #2 | TITLE-ABS-KEY (intersectional* OR {gender} OR "sex diff*" OR "sex inequalit*" OR {sex bias} OR {sex-specific} OR "sex factor*" OR {sex based} OR "sex disparit*" OR "sex factor*" OR "sex characteristic*" OR {sex related} OR sexe* OR {male versus female} OR {men versus women} OR {men and women} OR {women and men} OR {male and female} OR {female and male} OR "female identit*" OR "male identit*") OR TITLE ({women} OR {woman} OR {female} OR {man} OR {men} OR {male}) | 2,429,648 |
| #3 | TITLE-ABS-KEY (satisf* OR perspective* OR perception* OR experienc* OR narrative* OR wish* OR desire* OR need* OR attitude* OR {stories} OR {story} OR account* OR challeng* OR problem* OR barrier* OR obstacle* OR concern* OR belief* OR believ* OR consideration* OR {considering} OR {well-being} OR {meaning} OR "care goal*" OR (patient* W/2 goal*) OR {dignity} OR "meaning-making*") | 25,056,278 |
| #4 | TITLE-ABS-KEY ("thematic analys*" OR "content analys*" OR "focus group*" OR ethnograph* OR ethnograf* OR etnograf* OR photovoic* OR "field stud*" OR fieldwork* OR "field work*" OR phenomenolog* OR narration* OR narrative OR qualitative OR multimethodolog* OR "mixed method*" OR observation* OR "grounded theory" OR "audio recording*" OR "tape recording*" OR audiotape* OR ((semi-structured OR semistructured OR unstructured OR informal OR in-depth OR indepth OR face-to-face OR structured OR guide*) AND (interview* OR discussion* OR questionnaire*))) | 4,726,149 |
| #5 | #1 AND #2 AND #3 AND #4 | 1,025 |

## IBSS (ProQuest) History and Search Details March 15, 2023

| **Search** | **Query** | **Results** |
| --- | --- | --- |
| S1 | MAINSUBJECT.EXACT("Heart failure") OR ti,ab("heart failure*" OR "cardiac failure*" OR "heart decompensation" OR "myocardial failure*" OR "heart insuffic*" OR "myocardial insuffic*" OR "decompensatio cordis" OR "HfpEF" OR "HfrEF" OR "paroxysmal dyspnea" OR "cardiac asthma*" OR "cardiac edema*" OR "cardiac oedema*" OR "cardiac decompensation" OR cadiomyopath* OR "diastolic dysfunct*" OR "systolic dysfunct*") | 395 |
| S2 | MAINSUBJECT.EXACT("Intersectionality" OR "Gender differences" OR "Gender" OR "Gender equity") OR ti,ab(intersectional* OR "gender" OR "sex diff*" OR "sex inequalit*" OR "sex bias" OR "sex-specific" OR "sex factor*" OR "sex based" OR "sex disparit*" OR "sex factor*" OR "sex characteristic*" OR "sex related" OR sexe* OR "male versus female" OR "men versus women" OR "men and women" OR "women and men" OR "male and female" OR "female and male" OR "female identit*" OR "male identit*") OR ti("women" OR "woman" OR "female" OR "man" OR "men" OR "male") | 305,701 |
| S3 | MAINSUBJECT.EXACT("Quality of life" OR "Activities of daily living") OR ti,ab(satisf* OR perspective* OR perception* OR experienc* OR narrative* OR wish* OR desire* OR need* OR attitude* OR stories OR story OR account* OR challeng* OR problem* OR barrier* OR obstacle* OR concern* OR belief* OR believ* OR consideration* OR considering OR well-being OR meaning OR "care goal*" OR (patient* goal*) OR dignity OR "meaning-making*") | 1,606,233 |
| S4 | S1 AND S2 AND S3 | 15 |
